# First report of the dagger nematode *Xiphinema pachtaicum* on onion in Morocco

**DOI:** 10.21307/jofnem-2019-028

**Published:** 2019-05-27

**Authors:** Fouad Mokrini, Abdelfattah Dababat

**Affiliations:** 1 Nematology Laboratory, National Institute for Agricultural Research (INRA), UR-Integrated Crop Protection, Agadir, Morocco; 2 International Maize and Wheat Improvement Center (CIMMYT), Ankara, Turkey

**Keywords:** Morocco, *Xiphinema pachtaicum*, Detection, First report

## Abstract

In 2018, during a survey in Souss region of Morocco, the dagger nematode *Xiphinema pachtaicum* was found parasitizing onion, (*Allium cepa* L.). The populations of dagger nematode were identified on the basis of their morphological and morphometric characters and by sequencing and analysing the D2D2 region of 28S rRNA. Microscopic observation of females demonstrated the occurrence of *Xiphinema pachtaicum* on onion. The 28S D2D3 sequences shared 99 to 100% similarity with sequence corresponding to *X. pachtaicum* in GenBank. To our knowledge this is the first report of *Xiphinema pachtaichum* infecting onion in Morocco.

Nematodes of the genus *Xiphinema* are ectoparasites that feed on extensive range of hosts. Some species of this genus are economically important pests of agricultural plants and others are vectors of Nepovirus. In April 2018, during a survey, specimens of dagger nematode (*Xiphinema* spp.) were collected from soil around the rhizosphere of onion (*Allium cepa* L.) with poor growth appearance and low yield from Ouled Dahou, Souss-Massa region of Morocco (Fig. 1). Nematodes were extracted from soil using a modified Baermann technique (Hooper, 1990). On an average, nine nematodes per 100 cm^3^ soil were obtained. The collected nematodes were subjected to morphological and molecular characterization. All specimens were identified as *Xiphinema pachtaicum* based on the alpha-numeric polytomous identification key codes developed by Lamberti et al. (2002). The females had a body forming a close C after fixation. Lip region was distinctly offset by constriction. Odontostyle was robust and odontophore had weak flanges. Morphometric measurements of *Xiphinema pachtaicum* are listed in Table 1. The morphometric data of described Moroccan specimens were perfectly fit within the two populations of *Xiphinema pachtaicum* recorded by Orlando et al. (2016) from Italy.

**Table 1.** Morphometric measurements of *Xiphinema pachtaichum*.

Character	*Xiphinema pachtaicum* (Females)
*n*	10
L	1,822 ± 40.5 (1770–1891)
a	63 ± 2.4 (59–65)
b	6.3 ± 0.2 (5.9–6.7)
c	62 ± 4.6 (54–67)
c’	1.7 ± 0.2 (1.4–2)
V	53.8 ± 3 (49–58)
Lip region width	8.7 ± 0.4 (8.1–9.3)
Odontostyle	83 ± 3.5 (78–88)
Odontophore	49 ± 4.7 (44–57)
Pharynx	289 ± 12.5 (270–315)
Body width	28.9 ± 0.8 (28–30)
Anal body width	17.2 ± 1.2 (16–19)
Tail length	29.4 ± 2.1 (28–34)
Anterior end to vulva	979 ± 53 (891–1051)

Note: All measurements are in ?m, measurements presented as mean ± standard deviation (range).

**Figure 1:**
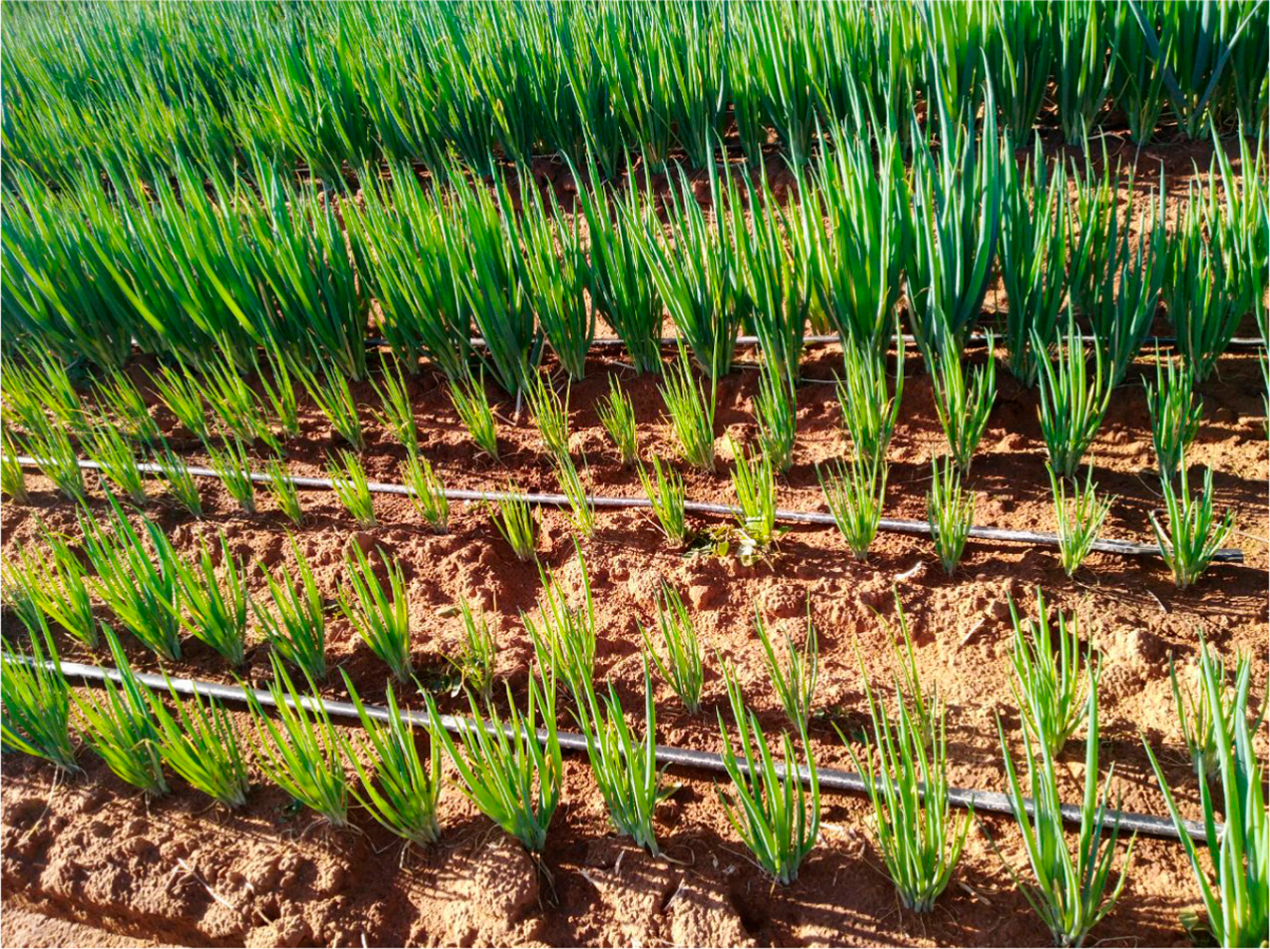
*Xiphinema pachtaicum* damage symptoms on onion plants include stunting of plants and yellowing of leaves.

To confirm the identity of *X. pachtaicum*, DNA was extracted from single females (*n* = 2) by using the protocol described by Holterman et al. (2006). Two primers were used: forward D2a (5′ ACAAGTACCGTGAGGGAAAGTTG 3′) and reverse D3b (5′ TGCGAAGGAACCAGCTACTA 3′) for the amplification of the D2D3 region of 28S rRNA (Nunn, 1992). The PCR products (represented by accession Nos. MK622911 and MK622912) were sequenced, aligned and compared with published sequences by means of BLAST search in the database. The comparison revealed 99 to 100% similarity, with sequence corresponding to *X. pachtaicum* and 97% or less sequence similarity with other *Xiphinema* spp. To our knowledge, this is the first report of *X. pachtaicum* parasitizing onion in Morocco.
